# 

**Published:** 1877-05

**Authors:** 


### THE OHIO DENTAL COLLEGE.

The announcement of this Institution is just issued, and will be sent to all who desire to know more about the College, if they will make this wish known to the Dean, or any member of the Faculty.

### MICHIGAN DENTAL SOCIETY.

The President, Dr. G. R. Thomas, has appointed the following Fellows of the Michigan Dental Association to fill the Standing Committees, viz:

ANATOMY AND HISTOLOGY.

D. C. Hawxhurst, Battle Creek. E. G. Douglass and G. W. Stone.

PHYSIOLOGY.

Prof. J. A. Watling, Ypsilanti. W. D. Tremper, and R. H. Tremper.

CHEMISTRY AND MATERIA MEDICA.

I. Douglass, Romeo. R. S. Bancroft and J. E. Post.

HYGIENE.

B. T. Spellman, Detroit. J. Lathrop and H. H. Jackson.

PATHOLOGY AND SEMEIOLOGY.

J. A. Robinson, Jackson. G. H. Mosher, and Thomas Rix.

THERAPEUTICS.

J. W. Finch, Adrian. M. H. Knapp and W. P. Morgan.

SURGERY.

D. W. Smith, Jackson. W. H. Jackson and E. Hunter.

EDUCATION AND ETIQUETTE.

E. S. Holmes, Grand Rapids. T. R. Perry and J. C. Parker.

PUBLISHING.

E. S. Holmes, Grand Rapids. G. R. Thomas, and W. D. Tremper.

### Executive.

G. L. Field, Detroit. J. A. Watling, and D. W. Smith.

With the President, Vice President, Secretary and Treasurer Ex-officio.

### Board of Censors.

Prof. J. A. Watling, for one year; D. C. Hawxhurst, for two years; W. H. Jackson, for three years.

### Consulting and Visiting.

G. R. Thomas, for one year; A. T. Metcalf, for two years; B. T. Spellman, for three years.

These gentleman are expected to be prepared, and to report, either in person or through the Chairman of their respective committees, on some point of the general subject assigned them, at the next session of the Michigan Dental Association, to be held at Ann Arbor on the 9th day of October, 1877, at 2 o'clock P. M.

The Secretary would respectfully suggest the following points for discussion by the several committees, viz: Anatomy etc. The Anatomy and Histology of the dental pulp.

*Physiology*—The provisions of nature for the protection of the dental pulp threatened with disease, and to what extent can they be relied on.

*Chemistry and Materia Medica*—The chemical constituents, and medical properties of Lacto Phosphate of Lime.

*Hygiene*—The prophylactic measures best adapted to prevent the consumption of the dental organs.

*Pathology and Semeiology*—The symtoms of dental exostosis, and the nature of the affection.

*Therapeutics*—The medicinal remedies for Dental Neuralgia.

*Surgery*—The surgical treatment of the Dental Pulp, diseased by the decomposition of the dentinal tissues.

There are other very important subjects to be considered at this meeting, and it is hoped and expected that every member of the association will be present, and prepared to do his duty.

A general invitation is hereby extended to all dentists—and every one interested in dental progress—to be present.

E. S. Holmes, *Sec'y M. D. A.*

### CONTENTS.

### COMMUNICATIONS.

### THE DENTAL REGISTER,

A MONTHLY MAGAZINE DEVOTED TO THE INTERESTS OF

**THE DENTAL PROFESSION.**

*TERMS REDUCED TO $2.50 PER YEAR. SINGLE COPIES 25C.*

EDITED BY PROF. J. TAFT, D.D.S.

AND

*Published by SPENCER, CROCKER & CO., Ohio Dental Depot.*

The volume commences in January and closes in December.

The REGISTER being issued monthly is always fresh, therefore Indispensable to the practitioner who desires to keep posted up with the new inventions and improvements which are daily developed. Neither expense nor effort will be spared to give value and variety to its contents. Articles will be illustrated with well executed engravings whenever they will add to the interest or assist to explanation.

Its extensive circulation and frequency of issue make it one of the BEST DENTAL ADVERTISING MEDIUMS in the United States.

All subscriptions must begin with January or July.

Local or special notices, five lines or less, will be inserted for $3 each insertion; over five lines, 50 cts. per line.

All advertising bills payable in advance, or at furthest, after the issue of the first number containing the advertisement. All transient advertisements to be paid for in advance.

☞CONTRIBUTIONS SOLICITED.

Exclusively Editorial Communications should be addressed to Editor.

The latest number of the REGISTER may be ordered with or without other goods at any time, and all letters should be addressed to

*SPENCER, CROCKER & CO., Ohio Dental Depot, Cincinnati, O.*

